# Aging in Context: The Importance of Comparative Studies for Understanding the Lives of Older Adults

**DOI:** 10.1093/geroni/igaa057.1847

**Published:** 2020-12-16

**Authors:** Allen Glicksman, Lauren Ring, Norah Keating

## Abstract

Access and use patterns of both formal and informal services for older migrants are often examined in a dyadic framework where one group of older persons (or their caregivers) is studied in relation to their use of a program or service. A comparative approach, that might also examine the reasons that some persons may (or may not) use a service, may yield important findings that place the dyadic studies within a larger social and policy context. By using a comparative approach, we can also consider influences of the culture of origin for older adults and their caregivers, as well as the policies and programs offered in the destination country. The four papers on this panel explore these issues. The first paper will frame the discussion, and the remaining three will focus on informal care, formal care, and the point of contact between aging services professionals and older immigrants. The first paper (Torres) takes a broad look at social exclusion mechanisms that bar access to services due to racism in the host societies. The second presentation (Diederich, et. al.) examines how place of origin can influence caregiving behavior. The third paper, (Thiamwong) examines a single program that is used to serve multiple ethnic minority/immigrant groups. Finally, (Ring et. al.,) will examine trust or its absence in the attitudes of older migrants toward use of formal aging services in two migrant populations. The four papers also demonstrate how different research methods (qualitative, quantitative, scoping review) can be used to illuminate these issues. International Aging and Migration Interest Group Sponsored Symposium.

